# What influences Australian pharmacists' decisions to supply opioids? Results from a survey and randomised controlled factorial experiment

**DOI:** 10.1111/dar.14009

**Published:** 2025-02-06

**Authors:** Louisa Picco, Ting Xia, Rose Laing, Elizabeth Grist, Jana Dostal, Suzanne Nielsen

**Affiliations:** ^1^ Monash Addiction Research Centre, Eastern Health Clinical School Monash University Melbourne Australia

**Keywords:** real‐time prescription drug monitoring program, PDMP, clinical decision‐making, opioids, pharmacists

## Abstract

**Introduction:**

Prescription drug monitoring programs (PDMP) identify medication‐related risks to support clinical decision‐making. This study aims to report prescription medication refusal rates based on PDMP alerts and determine how PDMP alerts and other characteristics influence pharmacists' decisions to supply opioids.

**Methods:**

Pharmacists completed an online survey and randomised controlled factorial experiment. Generalised linear models explored differences in the proportion of refusals by state. Pharmacists indicated their likelihood to supply opioids based on six clinical vignettes. Mixed‐effect linear regression models explored the association between vignette and pharmacy‐related characteristics and the likelihood to supply opioids.

**Results:**

Data related to 598 pharmacists (*n* = 3370 vignettes). Jurisdiction was associated with refusals; Western Australian pharmacists had significantly lower odds of refusing supply compared with Victorian pharmacists (odds ratio = 0.186, 95% confidence interval 0.104–0.331). The factorial experiment revealed the strongest predictors of reduced likelihood to supply were the PDMP high dose (β = −2.76, *p* < 0.001) and multiple prescriber (β = −3.79, *p* < 0.001) alerts. Unemployment (β = −0.0421, *p* < 0.001), hepatitis C (β = −0.260, *p* = 0.009), depression (β = −0.301, *p* = 0.003), high opioid dose (β = −0.259, *p* = 0.002) and co‐prescribed opioids with benzodiazepines (β = −0.478, *p* = 0.001) resulted in smaller reductions in the likelihood to supply. Older patient age, patient familiarity and rural/remote pharmacies were associated with significant, albeit small increases in the likelihood to supply with respective 0.293‐, 0.250‐ and 0.339‐unit increases.

**Discussion and Conclusions:**

Jurisdictional differences in refusal to supply were observed, while PDMP alerts were the strongest predictor of reduced likelihood to supply opioids in the factorial experiment. Unintended consequences of PDMPs including abrupt opioid discontinuation observed elsewhere, should be avoided following the implementation of this supply‐side policy in Australia.

## 1 INTRODUCTION

Increases in opioid prescribing in recent decades, coupled with long‐term and or non‐medical use can result in substantial harm including mortality and morbidity [1]. In the United States, the opioid epidemic was initially driven by prescription opioids, however, more recently, illicit opioids are the main contributing factor to high mortality rates [2]. In comparison, despite recent decreases in prescribing rates in Australia [3], rates per capita are higher in Australia compared to the United States [4]. To mitigate risks and reduce opioid‐related harm, various supply‐side policies have been adopted, with one of the most common being prescription drug monitoring programs (PDMP).

PDMPs have predominantly been implemented in North America and Australia, however, there is considerable differences in how these tools are operationalised, with limited research outside the United States on how they influence opioid supply [5]. In the United States, some PDMPs can be accessed by health‐care professionals and law enforcement and consequently have been described as a ‘policing’ tool for patients [6]. In Australia, PDMPs are a clinical risk management tool that capture individual patients' prescribing and dispensing history for controlled medicines such as opioids, in real‐time. They utilise computerised algorithms that trigger alerts, commonly relating to medication‐related risks such as high‐dose or high‐risk drug combinations and are designed to help inform clinical decision‐making [5]. More specifically, the information within PDMPs can help pharmacists decide whether it is safe and appropriate to supply medications, based on prescription histories and identified risks.

Pharmacists are responsible for the supply of prescription medications, ensuring that patients are educated on the safe and effective use of their medicines while minimising harm including medication misuse [7]. Given that pharmacists are required to assess the risks versus benefits when supplying high‐risk medicines, often in a time‐sensitive manner, a better understanding of how pharmacists respond to PDMP alerts and the factors that influence these clinical decisions is important to ensure optimal patient outcomes. It is also important that unintended consequences that have been observed in the United States including abrupt medication discontinuation, rapid tapering, treatment refusal, stigma and ‘policing’ patients are avoided [5, 8] in Australia. Therefore, combining clinical and professional judgements with more objective information within PDMPs may help better inform pharmacists' clinical decisions and avoid such unintended consequences.

The Australian government recently funded national prescription monitoring and, as of March 2023, all Australian jurisdictions have operational real‐time PDMPs, which are accessible to community pharmacists [9]. Little is known, however, about how implementation of this supply‐side policy in Australia influences clinical decisions relating to prescribing and dispensing practices, with most studies to date focusing on the state of Victoria's PDMP. For example, initial Victorian studies have shown PDMP implementation did not appear to influence or reduce the prescribing of high opioid doses, but did result in increases in the initiation of non‐monitored medications such as pregabalin, while PDMP alerts were a significant predictor of reduced likelihood to supply opioids among pharmacists [10, 11]. It is also unclear whether specific differences in these policies, including mandates to check the PDMP, influence clinical decision‐making, including decisions to supply medicines or not. To address these gaps, the current study aimed to: (i) explore differences in self‐reported medication refusal based on PDMP alerts among pharmacists; and (ii) determine how PDMP alerts and patient, pharmacy and medication‐related characteristics influence pharmacists' decisions to supply opioids, utilising vignettes within a randomised controlled factorial experiment.

## 2 METHODS

### 2.1 Design

An online anonymous survey capturing information on self‐reported supply refusal rates and incorporating a randomised controlled factorial experimental design was adopted. Factorial experiments allow researchers to better understand how variables of interest (e.g., PDMP alerts) impact participants' judgement or decisions, in this case, the decision to supply an opioid prescription. Ethical approval for the study was granted by Monash University Human Research Ethics Committee (No. 36459).

### 2.2 Setting

Pharmacists from four Australian states, New South Wales (NSW), Queensland, Victoria and Western Australia (WA), were invited to participate. These states were specifically selected as they are among the most populous Australian states, representing 88% of the Australian population [12] and they provide variation in terms of mandates to check the PDMP and the time in which PDMPs had been implemented [9].

Australian PDMPs utilise a traffic light (i.e., red, amber and green) notification and alert system to inform health‐care providers of the severity of possible medication‐related risks, based on medications prescribed and dispensed in the preceding 90 days. In most instances, the PDMP is integrated into prescribing or dispensing software, with pop‐up notifications appearing in real‐time as monitored medications are prescribed or dispensed. Prescribers and pharmacists then click on these notifications and are redirected to the PDMP where they can see a patient's prescribing and dispensing history for monitored medications. They are provided with information on the alert type and the reason which triggered the PDMP alert. While each Australian PDMP monitors varying risks, all four states included in this study have red high‐risk alerts relating to high dose and multiple prescriber episodes and green alerts which signify no risk according to the PDMP algorithm. For Queensland and Victoria, states where PDMP use is mandatory, prescribers and pharmacists are required to check the PDMP when prescribing or dispensing a monitored medicine where a red or amber notification appears in the prescribing or dispensing software, while in NSW and WA, use is voluntary, with no requirements to check the PDMP where alerts appear.

### 2.3 Sampling of pharmacies and sample size

Maven Marketing and Australian Marketing List pharmacy marketing lists were used to identify community pharmacies in each state. These lists were combined and duplicates and non‐community pharmacy listings were removed (see Figure S1, Supporting Information, for recruitment figures by state).

‘Raosoft’, an online sample size calculator [13] was used to calculate the minimum sample size, based on the total identified number of community pharmacies, confidence level of 99% and margin of error 5%. The sample calculation determined a minimum sample size of 588 was required to ensure a representative sample of pharmacies, hence we approached 1956 pharmacies, based on the response rate of a similar study [10]. The pharmacy list for each state was randomised using the Microsoft Excel formula ‘= rand()’ and a randomly selected subset within each state was then approached.

### 2.4 Participants

Individual pharmacies in each state were contacted via telephone and invited to participate in the online survey. The pharmacist in charge was invited to participate and, to avoid clustering effects, only one pharmacist per pharmacy was invited.

### 2.5 Procedures

The online survey was administered via Qualtrics. A total of 1956 pharmacies were approached between August and September 2023. Upon agreeing to participate over the phone, pharmacists provided an email address to which the online survey link was sent, as were follow up emails after 1 and 2 weeks from the initial email. Pharmacists were required to provide consent before commencing the survey and at the end of the survey, they had the option to enter a prize draw to win one of two iPads, which was captured in an unlinked secure portal.

### 2.6 Pharmacist and pharmacy related questions

Participants were asked to provide information on demographic and pharmacy‐related characteristics. Information pertaining to individual pharmacists included age, gender and years of pharmacy practice. Pharmacy‐specific questions related to the state in which the pharmacy was located, geographic location, pharmacy type (i.e., independent or part of chain or banner group) and opening hours, as well as services offered including stocking naloxone and offering opioid agonist treatment (i.e., methadone or buprenorphine).

### 2.7 Outcome measures

#### 2.7.1 Supply refusal

Pharmacists were asked to indicate on a 0–100% scale how often in the past 3 months they had refused supply, based on a PDMP alert.

#### 2.7.2 Randomised controlled factorial experiment

A series of vignettes were developed and tested as part of an earlier randomised controlled factorial study and the processes of incorporating clinical inputs and testing of the vignettes with pharmacists are described elsewhere [10].

Briefly, vignettes comprised of a total of eight patients, medication and PDMP‐related variables or characteristics known to influence clinical decision‐making, with each variable having between two and four levels (see Table S1 and Figure S2). The PDMP variable was worded similarly to those actual alerts that would appear in Australian PDMPs. Vignettes were structured similarly, however, the associated levels (e.g., high dose alert, multiple prescriber alert, no PDMP alert) for each variable were experimentally varied, enabling the examination of variables on supply decisions [14, 15]. A total of 72 versions of the vignette were used, where pharmacists were randomised to 1 of 12 blocks of six vignettes with the order of these vignettes also being randomised to avoid order effects. After reading the vignettes, pharmacists were asked to indicate how likely they were to supply the opioid using a 0 (not at all likely) to 10 (extremely likely) scale.

### 2.8 Statistical analysis

Descriptive statistics were used to summarise pharmacy characteristics. The mean, median and the interquartile range value were reported for the proportion of refusals to supply in the past 3 months based on a PDMP alert. To further explore the differences in the proportion of refusals by state, generalised linear model with a logit link and the binomial family was used. Values above the 99th percentile with the upper limit were removed to eliminate outliers and ensure a more accurate analysis. The final model was adjusted for pharmacy location and type.

Mixed‐effect linear regression analyses were adopted to explore the association between independent variables (refer to Tables S1 and 1, respectively) and the dependent variable (likelihood to supply from 0 to 10). The intraclass correlation was calculated based on the fit of a random‐intercept only model (null model), with no predictor variables. Subsequently, vignette and pharmacy‐level variables were added sequentially in two steps, and changes in the intraclass correlation were compared. Likelihood ratio tests were used to measure the goodness of model fit and identify the most parsimonious model. A coefficient unit change in the likelihood to supply and 95% confidence interval (CI) were used to measure the effect of each variable. Statistical analyses were conducted using Stata 17SE with two‐sided *p*‐values <0.05 considered statistically significant.

**TABLE 1:** Sample characteristics.

Characteristics	*N* (%)
Gender	
Female	295 (49.3)
Male	299 (50.0)
Non‐binary/other	4 (0.6)
Age, years	
21–30	142 (23.8)
31–40	232 (38.8)
41–50	125 (20.9)
51–60	65 (10.9)
>60	34 (5.7)
Years of practice	
<15 years	360 (60.2)
≥15 years	238 (39.8)
Pharmacy state	
Victoria	164 (27.4)
New South Wales	138 (23.1)
Queensland	173 (28.9)
Western Australia	123 (20.6)
Pharmacy location	
Capital city	320 (53.5)
Urban	101 (16.9)
Rural and remote	177 (29.6)
Pharmacy type	
Independent pharmacy	192 (32.1)
Chain or banner group/other	398 (66.6)
Intercept	8 (1.3)
Pharmacy open days	
7 days	368 (61.5)
6 days	201 (33.6)
5 or less days	29 (4.9)
Pharmacy open hours	
Business hours	467 (78.1)
Extended hours	131 (21.9)

## 3 RESULTS

### 3.1 Sample characteristics

In total 1396 pharmacists were sent the survey link, of which data were collected from 598 and are reported in the current study, representing a response rate of 43%. Overall, half (*n* = 295, 49.3%) were female, and most pharmacists were aged 40 or less (*n* = 374, 62.6%) and had less than 15 years of pharmacy practice experience (*n* = 360, 60.2%) (Table 1). Most pharmacies were located in capital cities (*n* = 320, 53.5%), and were open 7 days per week (*n* = 368, 61.5%), while about one in five pharmacies were open extended hours (*n* = 131, 21.9%). Most pharmacies in all states reported dispensing opioids at least ‘several times a day’ (NSW 70%, Queensland 87%, Victoria 72%, and WA 69%).

### 3.2 Recent refusal to supply based on PDMP alerts

State differences in self‐reported refusal to supply based on PDMP alerts, (as measured by 0–100% scale in the past 3 months) were observed. On average, Victorian pharmacists reported that they refused to supply based on PDMP alerts 16.2% (SD = 22.1, median = 8 [interquartile range: 3, 19]) of the time in the past 3 months, NSW‐based pharmacists refused 14.5% (SD = 22.3, median = 5 [interquartile range: 1, 15]), Queensland pharmacists refused 14.4% (SD = 18.6, median = 9 [interquartile range: 3, 20]), while pharmacists in WA refused supply 3.5% (SD = 9.9, median = 0 [interquartile range: 0, 2]) in the past 3 months. Results from the generalised linear model revealed that pharmacists in WA had significantly lower odds of refusing to supply based on PDMP alerts compared with pharmacists in Victoria (OR = 0.186, 95% CI 0.104–0.331) (Table 2).

**TABLE 2:** Generalised linear model on the % of refusal.

Pharmacy characteristics	OR	95% CI	*p*‐value
Pharmacy state			
Victoria	Ref		
New South Wales	0.930	0.623, 1.387	0.721
Queensland	0.880	0.616, 1.257	0.482
Western Australia	0.186	0.104, 0.331	**0.0001**
Pharmacy location			
Capital city	Ref		
Urban	0.908	0.609, 1.354	0.551
Rural and remote	0.691	0.495, 0.967	**0.031**
Pharmacy type			
Independent pharmacy	Ref		
Chain or banner group/other	1.124	0.807, 1.567	0.487
Intercept			

*Note*: Bold indicates significant values *p* < 0.05.

Abbreviations: CI, confidence interval; OR, odds ratio; Ref, reference group.

### 3.3 Randomised controlled factorial experiment

A total of 3370 vignettes were included in the analyses. Most pharmacists (*n* = 528) completed questions about all six randomly assigned vignettes, with the remaining 70 providing data relating to between one and five vignettes. The likelihood ratio test revealed that Model 3, which included both the vignette and pharmacy variables had the best model fit (likelihood ratio 40.8, *p* < 0.001).

### 3.4 Likelihood to supply prescription opioids

Table 3 reports the predictors of the likelihood to supply opioid prescriptions on an 11‐point (0–10) scale and reveals a combination of vignette and pharmacy characteristics that significantly increased or decreased the decision to supply opioids. Vignette variables that increased the likelihood to supply included older age (i.e., 55 years [0.24 unit increase, 95% CI 0.062, 0.422; *p* = 0.008] and 75 years [0.29 unit increase, 95% CI 0.015, 0.571; *p* = 0.039]) when compared to 35 years, and being a regular pharmacy patient, which was associated with a 0.25 unit increase in the likelihood to supply (vs. a new pharmacy patient, 95% CI 0.113, 0.358; *p* < 0.0001). Pharmacy location was also a significant predictor of increased likelihood to supply; those in rural or remote locations observed a 0.38 unit increase in likelihood to supply when compared to pharmacies located within capital cities (95% CI 0.029, 0.649; *p* = 0.032).

**TABLE 3:** Predictors of the likelihood of supplying an opioid prescription.

	Coefficient	95% CI		*p*‐Value
**Vignette characteristics**				
Gender				
Female	Ref			
Male	−0.002	−0.139	0.136	0.980
Age				
35 years old	Ref			
55 years old	0.242	0.062	0.422	**0.008**
75 years old	0.293	0.014	0.571	**0.040**
Employment				
Employed	Ref			
Retired	−0.147	−0.368	0.074	0.192
Unemployed	−0.421	−0.602	−0.239	**<0.0001**
Familiarity				
New	Ref			
Regular	0.250	0.113	0.388	**<0.0001**
Prescribed opioid				
Oxycodone CR 10 mg twice daily	Ref			
Oxycodone CR 20 mg in the morning and 30 mg at night	−0.237	−0.406	−0.069	**0.006**
Oxycodone CR 40 mg twice daily	−0.259	−0.427	−0.092	**0.002**
Prescribed benzodiazepine				
Diazepam 5 mg 4 times a day	−0.478	−0.614	−0.342	**<0.0001**
No	Ref			
Co‐morbidity				
Hepatitis C	−0.260	−0.455	−0.064	**0.009**
Major depressive disorder	−0.301	−0.496	−0.106	**0.003**
Chronic obstructive pulmonary disease	−0.167	0.363	−0.028	0.093
No other chronic conditions	Ref			
Alert reason				
Red multiple prescriber alert	−3.791	−3.958	−3.624	**<0.0001**
Red high dose alert	−2.762	−2.931	−2.593	**<0.0001**
Green alert (no risk according to PDMP)	Ref			
Pharmacy characteristics				
Pharmacy state				
Victoria	Ref			
New South Wales	−0.733	−1.114	−0.351	**<0.0001**
Queensland	0.307	−0.059	0.672	0.100
Western Australia	−0.392	−0.786	0.001	0.051
Pharmacy location				
Capital city	Ref			
Urban	0.307	−0.081	0.696	0.121
Rural and remote	0.339	0.029	0.649	**0.032**
Pharmacy type				
Independent pharmacy	Ref			
Chain or banner group/other	0.007	−0.285	0.300	0.961
Intercept				0.000

*Note*: Bold indicates significant values *p* < 0.05.

Abbreviations: CI, confidence interval; CR, controlled‐release; OR, odds ratio; PDMP, prescription drug monitoring program; Ref, reference group.

A range of vignette variables also significantly decreased the likelihood to supply. The strongest predictors were the PDMP alerts, where a high dose alert was associated with a large (2.8 unit) reduction in the likelihood to supply (95% CI −2.931, −2.593; *p* = 0.001), while the multiple prescriber alert was associated with a 3.8 unit reduction in the likelihood to supply (95% CI −3.958, −3.624; *p* = 0.001), compared to the green alert (indicating no risk according to the PDMP algorithm). Other variables significantly associated with decreased likelihood to supply included employment status, with a 0.42 unit decrease for those that were unemployed, compared to those who were employed (95% CI −0.602, −0.239; *p* < 0.001), higher opioid doses (i.e., oxycodone controlled‐release [CR] 20 mg in the morning and 30 mg at night [0.24 unit reduction, 95% CI −0.406, −0.069; *p* = 0.006] and oxycodone CR 40 mg twice daily [0.26 unit reduction, 95% CI −0.427, −0.092; *p* = 0.002]), when compared to a lower dose of 10 mg oxycodone CR twice daily, a co‐prescription of opioids and benzodiazepines which saw a 0.48 unit reduction compared to those not prescribed benzodiazepines (95% CI −0.614, −0.342; *p* < 0.001) and a co‐morbidity of Hepatitis C (0.26 unit reduction, 95% CI −0.455, −0.064; *p* = 0.009) and major depressive disorder (0.30 unit reduction, 95% CI −0.0496, −0.106; *p* = 0.003), when compared to having no other chronic conditions. Pharmacy state was also associated with a reduced likelihood to supply; when compared to Victoria, there was a 0.73 unit reduction in the likelihood to supply in NSW (95% CI −1.114, −0.351; *p* < 0.001).

## 4 DISCUSSION

This study explored the effect of PDMP implementation on opioid supply through self‐reported refusal to supply after receiving actual PDMP alerts, and likelihood to supply, based on clinical vignettes as part of a randomised controlled factorial experiment. It is the first Australian study to report differences in PDMP‐related opioid supply effects across jurisdictions. It has a range of implications, given the recent implementation of this opioid policy in Australia, and adds to the limited PDMP literature originating outside of the United States.

Overall, there was evidence from all states that PDMP alerts resulted in the refusal to supply monitored medications for between 3% and 16% of instances. Pharmacists in WA were significantly less likely to report supply refusal based on PDMP alerts, compared with Victorian‐based pharmacists, with no difference between Victoria, NSW and Queensland. The lower likelihood of self‐reported refusal may be due to the more recent (past 6‐month implementation) period in which the WA PDMP had been in operation. Another possible explanation could relate to the voluntary nature of this PDMP which may have also resulted in reduced use of the PDMP and lower exposure to alerts, highlighting the importance of implementation and system factors on supply and refusal decisions. A pre‐implementation study among WA pharmacists revealed that many were not prepared for their state's PDMP implementation and required specific PDMP training, which may further explain the current finding [16]. Conversely, it could reflect recent training where pharmacists in WA had a more nuanced response to the alerts including contacting the prescriber or providing patient education, which then resulted in reduced refusals.

Findings from the randomised controlled factorial experiment revealed PDMP alerts were the most significant predictor of reduced likelihood to supply, which corroborates that of a similar study among Victorian pharmacists [10]. Both the frequency of actual supply refusal and the factors that contribute to the increased likelihood of refusing supply are important to understand given that abrupt refusal or discontinuation of a patient's medications is not recommended [17] and can result in various unintended consequences including unmanaged pain and opioid withdrawal [18], increased risk of overdose death [19], suicide and self‐harm [20, 21], and illicit opioid use [22, 23]. Our study appears to be one of the first in Australia to confirm that PDMP use is leading to pharmacists' refusing to supply opioids. This in turn highlights the need to understand factors that may decrease the likelihood for pharmacists to supply patients their prescribed opioids, which is also determined in this study.

While at times in the United States, PDMPs have been used as a policing tool to identify ‘bad’ patients [24], in Australia they have been promoted as a clinical risk management tool [25], and are intended to enable more informed therapeutic decisions, by identifying patients who may be at increased risk of medication‐related harm. That being said, an Australian study found that for some health‐care providers, PDMP alerts are synonymous with dependence or addiction [26] and therefore such ‘policing’ practices may also be occurring locally, however, given the low refusal rates, this is thought to be less common. Clinical guidelines to support health‐care providers on how to interpret and respond to PDMP information are currently lacking [27] and may assist pharmacists to better respond to PDMP alerts, while mitigating further risk and improving patient outcomes. The development of PDMP‐related clinical guidelines should be a future priority given the increasing adoption of this opioid policy internationally. Furthermore, there appears to be a need for widescale healthcare provider education to reinforce that PDMP alerts only identify medication‐related risks, and should not replace clinical judgement on the need for medications.

Although PDMP alerts appeared to be the largest driver of supply refusal, other well‐established clinical risk factors including high opioid dose and the concurrent use of opioids and benzodiazepines were also associated with reduced likelihood to supply, albeit a 10‐fold smaller magnitude. This may reflect various recent initiatives including prescribing and deprescribing guidelines that caution the co‐prescription of opioids and benzodiazepines and the prescribing of high opioid doses [28, 29]. Because PDMPs alert health‐care providers of high‐risk drug combinations such as these, the recent implementation of PDMPs in Australia may reflect pharmacists' increased awareness of these high‐risk drug combinations and explain the reduced likelihood to supply.

Hepatitis C and Major Depressive Disorder were also associated with a reduced likelihood to supply opioids when compared to having no comorbidity. This may represent a concerning finding as these comorbidities would not necessarily suggest that opioids should not be supplied and therefore may reflect possible stigma. The vignette which describes a patient with Hepatitis C also refers to injecting drug use, which may be a contributing factor to the reduced likelihood to supply [30], with existing research revealing pharmacists hold stigmatising views about injecting drug use [31]. Similarly, mental illnesses such as depression and poorer socio‐economic circumstances including unemployment are also highly stigmatised [32] and approximately one in two people with chronic pain also have depression [33]. An existing systematic review found PDMP use resulted in increased stigmatising clinical responses, including using these clinical tools to ‘police’ or ‘weed out problem patients’ [5]. Stigmatising views held by pharmacists may not only impact patient care and contribute to greater health inequalities [34] but also appear to influence clinical decision‐making around a reduced likelihood of supplying opioids to these vulnerable populations. The current findings suggest the need for future research and stigma‐specific interventions and training for pharmacists.

Some vignette characteristics were associated with an increased likelihood to supply, including older age and patient familiarity. Given that chronic pain and the use of prescription opioids are more common among older adults (>55 years) [35, 36], this is likely to explain pharmacists' increased comfort and likelihood to supply opioids to older patients, compared to those aged 35 years. Patient familiarity, which is likely to encompass factors including a patient's medical history, indication for the prescribed opioid, current medications, and related doses is likely to explain pharmacists' increased likelihood to supply opioids to regular pharmacy patients, compared to new patients. A recent study among Australian prescribers and pharmacists also described factors such as patient familiarity influencing how and when they used PDMPs [37].

When considering self‐reported refusal rates, pharmacists in WA appeared the least likely to refuse supply, though when examined in experimental vignettes, the likelihood was also less compared to Victoria, however, just failed to reach statistical significance (*p* = 0.051). The factorial experiment did find, however, that pharmacists in NSW were less likely to supply compared to those in Victoria. Although we cannot confirm why this is the case, it could be attributed to variability in the PDMP programs or the duration in which they have been in operation. For example, Victoria's PDMP has been in operation longer and therefore pharmacists may experience possible alert fatigue, paying less attention or placing less emphasis on PDMP alerts [38]. Or it could relate to mandatory requirements for Victorian pharmacists to check the PDMP before dispensing, unlike pharmacists in NSW, which result in increased familiarity with alerts with an associated increased confidence to supply even when a high‐risk alert is triggered. Research exploring jurisdictional differences including nuances of PDMPs which may enhance patient outcomes is warranted [39]. Findings may also reflect jurisdictional differences in opioid prescribing requirements and rates of opioid use or misuse [40, 41, 42]. Pharmacies located in rural or remote locations had an increased likelihood of supplying opioids, compared to those in capital cities. This finding may be related to higher rates of opioid utilisation in rural areas [43], where pharmacists in these geographic locations are more exposed to opioid prescriptions and are therefore more comfortable and likely to supply.

### 4.1 Strengths and limitations

This is the largest study exploring Australian pharmacists' decision‐making to supply opioids, following the implementation of PDMPs in Australia. All community pharmacists had access to their jurisdictions' PDMP at the time data were collected, with included pharmacies representing 11.2% of all Australian pharmacies, and yielded a response rate of 43%. The study captured self‐reported refusal based on PDMP alerts and utilised established clinical vignettes, as part of a rigorous randomised controlled factorial experimental design, a widely adopted methodology in social research, particularly about judgements, attitudes, beliefs and perceptions [14]. The following limitations should be considered when interpreting the findings. Social desirability and recall bias may have impacted responses to certain questions. It is possible that a small number of Victorian pharmacists were randomly selected and participated in the current and an earlier study, which utilised a similar design, however, due to random selection and high pharmacist turnover this is not likely to have had a major effect. Furthermore, there were several years between the two studies, and pharmacists were never provided with answers to the questions, thus decreasing the likelihood of their responses having previous influence.

## 5 CONCLUSION

PDMPs are a widely adopted supply‐side opioid policy designed to reduce opioid‐related harms, where alerts influenced self‐reported decisions to refuse opioid supply, with state differences being identified. Both self‐reported refusal rates and the randomised controlled experiment utilising clinical vignettes revealed PDMP alerts were associated with meaningful changes in the likelihood to supply, especially when considered relative to other established clinical risk factors. This is an important finding given the potential unintended consequences for patients if supply is abruptly discontinued. Factors associated with economic disadvantage and stigma were also associated with a reduced likelihood to supply. It is important that unintended consequences, particularly those observed in North America, including medication refusal or rapid tapering, are avoided following the implementation of this supply‐side policy in Australia.

## AUTHOR CONTRIBUTIONS

**Louisa Picco**: Conceptualisation, Methodology, Investigation, Data curation, Writing – Original Draft, Supervision, Project Administration, Funding Acquisition. **Ting Xia**: Formal analysis, Data curation, Writing – Review and Editing. Rose Laing: Investigation, Writing – Review and Editing. **Elizabeth Grist**: Investigation, Writing – Review and Editing. **Jana Dostal**: Investigation, Writing – Review and Editing. **Suzanne Nielsen**: Writing – Review and Editing, Supervision.

## CONFLICT OF INTEREST STATEMENT

The authors have no conflicts to report.

## Supporting information


**Data S1.**Supporting information.

## Data Availability

The data that support the findings of this study are available on request from the corresponding author. The data are not publicly available due to privacy or ethical restrictions.
