# Supplementary material for: What influences Australian pharmacists' decisions to supply opioids? Results from a survey and randomised controlled factorial experiment
Source: Drug Alcohol Rev. 2025 Feb 6;44(3):783–92. doi: 10.1111/dar.14009 (PMC11886474; doi:10.1111/dar.14009)
Supplement: Supplementary file 1 — Data S1.Supporting information. [file DAR-44-783-s001.docx]

.

n=598 pharmacists form the final dataset

n=92 were excluded due to not answering question on vignettes

n=706 pharmacists did not commence/complete minimum data requirement

n=690 pharmacists participated in the survey

n=1396 pharmacists were sent the survey link for participation

Excluded cases n=559

n=286 were uncontactable

n= 58 not community pharmacies

n= 20 were duplicates

n= 66 had not used state PDMP

n= 3 completed survey at another pharmacy

n= 126 pharmacists declined participation over the phone
(n= 59 due to no time and
n= 67 were not interested)

Subset of n=1955 pharmacies identified via marketing lists (n= 500 NSW; n=500 Queensland, n= 502 Victoria, n=453 Western Australia)

#

**Figure S1: Flow diagram of participation. NSW, New South Wales; PDMP, prescription drug monitoring programs.**

**Table S1: Vignette-level variables**

| Variables | Levels |
| --- | --- |
| Gender (of patient) | - Male - Female |
| Age (of patient) | - 35 years old - 55 years old - 75 years old |
| Employment (of patient) | - Employed - Unemployed - Retired |
| Familiarity (of patient to pharmacist) | - Regular - New |
| Prescribed opioid | - Oxycodone CR 10 mg twice daily - Oxycodone CR 20 mg in the morning and 30 mg at night - Oxycodone CR 40 mg twice daily |
| Prescribed benzodiazepine | - Also taking Diazepam 5 mg 4 times a day for his/her back pain. This is prescribed by the same doctor who prescribed the oxycodone. - Not taking any other sedative medicines. |
| Co-morbidity | - Chronic obstructive pulmonary disease (and are currently using a fluticasone inhaler to treat this. - A current diagnosis of major depressive disorder which is being treated and is stable, and they not taking any current medicines for this - Hepatitis C, as a result of injecting drug use in the past, but has just finished treatment and waiting to find out if he/she has cleared the virus. The doctor has confirmed his/her liver function is fine. - No other chronic conditions. |
| PDMP alert reason* | - SafeScript indicates a red alert informing you he/she is receiving opioids exceeding 100 mg morphine equivalent dose daily in the last 90 days. Her history shows oxycodone has been prescribed by the same doctor and there have been several recent prescriptions for oxycodone. - SafeScript indicates a red alert informing you he/she has obtained prescriptions for SafeScript monitored medicines from at least 4 different prescribers in the last 90 days. - SafeScript indicates he/she has only had monitored medicines prescribed by the single prescriber and there are no alerts or other recent opioid dispensing (green alert). |

CR, controlled-release; PDMP, prescription drug monitoring program.

* Example here relates to Victoria’s PDMP ‘SafeScript’

**Figure S2:** **Example of vignette skeleton with randomly populated vignette**

**Vignette skeleton**

- [Gender], [age], [employment] [familiarity]
- Comes in with a script for [prescribed opioid] which has been prescribed for 6 months.
- He/she is [prescribed benzodiazepine]
- He/she informs you he/she has [co-morbidity]
- The prescription drug monitoring program indicates [alert reason]

**Example vignette**

Female, 35 years old, employed, regular patient at your pharmacy

Comes in with a script for oxycodone controlled-release 40 mg twice daily for chronic pain, which has been prescribed for 6 months.

She is not taking any other sedative medicines.

She has a current diagnosis of major depressive disorder which is being treated and is stable, and they not taking any current medicines for this.

SafeScript indicates a red alert informing you she is receiving opioids exceeding 100 mg morphine equivalent dose daily in the last 90 days. Her history shows oxycodone has been prescribed by the same doctor and there have been several recent prescriptions for oxycodone.
